# Are Current Atomistic Force Fields Accurate Enough to Study Proteins in Crowded Environments?

**DOI:** 10.1371/journal.pcbi.1003638

**Published:** 2014-05-22

**Authors:** Drazen Petrov, Bojan Zagrovic

**Affiliations:** Max F. Perutz Laboratories, University of Vienna, Vienna, Austria; Fox Chase Cancer Center, United States of America

## Abstract

The high concentration of macromolecules in the crowded cellular interior influences different thermodynamic and kinetic properties of proteins, including their structural stabilities, intermolecular binding affinities and enzymatic rates. Moreover, various structural biology methods, such as NMR or different spectroscopies, typically involve samples with relatively high protein concentration. Due to large sampling requirements, however, the accuracy of classical molecular dynamics (MD) simulations in capturing protein behavior at high concentration still remains largely untested. Here, we use explicit-solvent MD simulations and a total of 6.4 µs of simulated time to study wild-type (folded) and oxidatively damaged (unfolded) forms of villin headpiece at 6 mM and 9.2 mM protein concentration. We first perform an exhaustive set of simulations with multiple protein molecules in the simulation box using GROMOS 45a3 and 54a7 force fields together with different types of electrostatics treatment and solution ionic strengths. Surprisingly, the two villin headpiece variants exhibit similar aggregation behavior, despite the fact that their estimated aggregation propensities markedly differ. Importantly, regardless of the simulation protocol applied, wild-type villin headpiece consistently aggregates even under conditions at which it is experimentally known to be soluble. We demonstrate that aggregation is accompanied by a large decrease in the total potential energy, with not only hydrophobic, but also polar residues and backbone contributing substantially. The same effect is directly observed for two other major atomistic force fields (AMBER99SB-ILDN and CHARMM22-CMAP) as well as indirectly shown for additional two (AMBER94, OPLS-AAL), and is possibly due to a general overestimation of the potential energy of protein-protein interactions at the expense of water-water and water-protein interactions. Overall, our results suggest that current MD force fields may distort the picture of protein behavior in biologically relevant crowded environments.

## Author Summary

Protein behavior is strongly affected by highly crowded and interaction-rich environments, i.e., typical conditions in both biologically relevant systems, such as the cellular interior, and solution-based structural experiments, including NMR and different spectroscopies. On the other hand, primarily because of limited computational power, molecular dynamics (MD) simulations, a premier high-resolution method for analyzing structure, dynamics and interactions of proteins, have been predominantly used to study individual proteins at infinite dilution. To fill this gap, we use MD simulations to study the behavior of wild-type (aggregation-resistant) and oxidatively damaged (aggregation-prone) forms of villin headpiece at high concentration, and reveal unexpected limitations and inaccuracies of modern-day MD force fields when it comes to modeling proteins at physiologically or experimentally relevant concentrations.

## Introduction

Different macromolecules occupy up to 40% of the total cytoplasmic volume in typical cells, with proteins being the most abundant class of molecules [1], [2]. Importantly, such densely packed environments strongly affect various thermodynamic and kinetic properties of proteins including their structural stabilities, intermolecular binding affinities and enzymatic rates [3], [4]. Most drastically, as a consequence of high intracellular concentration, proteins can form cytotoxic aggregates that have been linked with numerous pathologies [5], [6]. Additionally, most solution-based biophysical experimental methods, such as NMR or different spectroscopies, involve samples with relatively high protein concentration. For these reasons, increasing attention has recently been devoted to studying protein behavior in crowded environments. For example, volume-exclusion and confinement effects in the context of crowding have been qualitatively well-understood by statistical mechanical theories and computer simulations [4], [7]. Moreover, Brownian dynamics and coarse-grained simulations have been used to provide a detailed description of multipart mixtures of biomolecules and have sometimes even matched real systems in their complexity [8]–[10]. Although highly successful, however, such approaches still fall short of capturing the fully atomistic, dynamic picture of high-concentration macromolecular systems. To this end, molecular dynamics (MD) simulations, a high-resolution computational biology tool [11], have recently been employed to model different aspects of crowding when it comes to protein structure, dynamics and interactions as well as solvent behavior [12]–[16]. Moreover, MD simulations have been applied to explore early events in the formation of protein aggregates, focusing predominantly on short peptides with high aggregation propensity [17]–[22]. On the other hand, arguably due to high computational expenses, control tests to show that non-aggregating polypeptides do not aggregate are rarely performed, with only a few attempts in this direction. For example, Gsponer *et al.* [17] and Tsai *et al.* [20] have shown that control mutant peptides aggregate into structures with reduced amyloid character as compared to aggregation-prone peptides. However, to the best of our knowledge, not a single study so far has provided clear evidence of a known non-aggregating polypeptide as a negative control.

Here, we use classical MD simulations to study the behavior of the 36-residue villin headpiece mini-protein [23]–[25] at atomistic resolution with multiple copies of the protein in the simulation box. In contrast to other MD simulation studies of protein-protein interactions or aggregation, our choice of the model system is primarily motivated by the fact that villin headpiece does *not* self-associate or aggregate at moderate protein concentrations. For example, this well-studied actin-binding polypeptide remains fully soluble at protein concentration of 1–2 mM as shown by infrared spectroscopy [26], [27]. Additionally, Fourier transform infrared spectra of the peptide indicate no aggregation at 6 mM when suspended in a 50 mM sodium acetate buffer with a possibility of aggregate or dimer formation only at a higher protein concentration of ∼18 mM [25]. Similarly, circular dichroism experiments suggest that there is no significant aggregation of villin headpiece at the concentration of 9.2 mM, although here the data was collected at −40°C in glycerol/water solution [24]. Finally, NMR spectra of villin headpiece can successfully be recorded even at the concentration of 32 mM, although with changes in chemical shifts of surface residues as a consequence of increased protein-protein interactions upon crowding [28].

As a control for our simulations of wild-type, folded villin headpiece, we also simulate the fully carbonylated, unfolded and aggregation-prone villin headpiece at high concentration. Increased levels of protein aggregation have been repeatedly related to protein oxidative damage with highly oxidized proteins being a frequent component of potentially cytotoxic aggregates [29]–[31]. In a recent study, we have shown that metal-catalyzed carbonylation, arguably one of the most important types of irreversible oxidation, drastically increases the intrinsic aggregability of villin headpiece by directly affecting its hydrophobicity, net charge and secondary structure [32], protein properties shown to strongly influence aggregation propensity [33]. Here, we study wild-type and carbonylated villin headpiece at experimentally relevant protein concentrations (6 mM and 9.2 mM) and different sodium chloride concentrations (0 to 0.8 M) using two different force fields (GROMOS 45a3 [34] and GROMOS 54a7 [35]), SPC water model [36] and two types of electrostatics treatment (PME - particle mesh Ewald [37] and RF - reaction-field [38]). Moreover, in order to account for potential inaccuracies in the physical description of the system, we use four additional force fields (AMBER94 [39], AMBER99SB-ILDN [40], CHARMM22-CMAP [41] and OPLS-AAL [42]) to reanalyze the wild-type villin headpiece GROMOS trajectories and employ two of these (AMBER99SB-ILDN and CHARMM22-CMAP) in combination with the TIP3P water model [43] to perform additional MD simulations. The principal question that we ask is how does the behavior of villin headpiece differ at high protein concentration as compared to infinite dilution and, in the process, we explore the limitations of current atomistic force fields in describing biologically and experimentally realistic protein solutions.

## Results

### Structural analysis of wild-type and carbonylated villin

**Figure 1:**
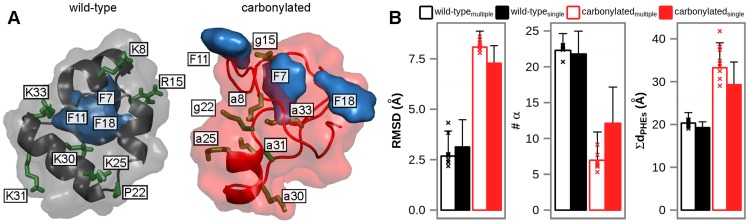
Structural characterization of villin headpiece in the wild-type folded state and the carbonylated unfolded state. A) typical structures of wild-type and carbonylated villin headpiece showing the core phenylalanines in blue and carbonylated residues in green; B) three measures of villin headpiece foldedness averaged over all trajectories simulated using GROMOS 45a3 and 54a7 force fields and given with standard deviations (data for infinite dilution taken from ref [32]). The averages were calculated over all GROMOS trajectories of finite protein-concentration systems, whereas only the last 25 ns of simulated time were used for infinite dilution systems.

Villin headpiece is a 3-helix bundle protein with a tightly packed hydrophobic core comprised of three phenylalanine residues (Fig. 1A) [23]. In order to structurally characterize the simulated proteins, we use: 1) atom-positional backbone root-mean-square deviation (RMSD) from the native experimental villin headpiece structure [23], 2) the number of residues in α-helical conformation (#α), and 3) the sum of distances between the centers of mass of core phenylalanines (Σd_PHEs_). In Figure 1B, we present aggregate averages of these three measures over all simulations performed using GROMOS 45a3 and 54a7 force fields for wild-type and carbonylated villin headpiece, with the average values for all individual simulation protocols given in Table S1. If not stated otherwise, these GROMOS simulations are used for analysis throughout this study. Note also that the initial configurations for simulations with multiple copies of the protein in the simulation box were chosen randomly from the equilibrium ensembles of the last 25 ns of the previously simulated 110-ns-long, infinite dilution trajectories of wild-type and carbonylated villin headpiece [32]. These same ensembles were also used for analysis of protein behavior at infinite dilution, thus ensuring that the two sets of simulations are fully comparable. Overall, wild-type polypeptides remain folded at infinite dilution as previously shown [32] with <RMSD> = 2.7±1.2 Å, <#α> = 22.3±2.3 and <Σd_PHEs_> = 20.3±2.4 Å (Fig. 1B, *wild-type_single_*). On the other hand, carbonylated molecules at infinite dilution populate the unfolded state [32], with an increase in the average RMSD (<RMSD> = 8.1±0.8 Å) and Σd_PHEs_ (<Σd_PHEs_> = 33.3±5.8 Å) and a significant decrease in the α-helical content (<#α> = 7.0±3.9) (Fig. 1B, *carbonylated_single_*). Importantly, both wild-type and carbonylated villin headpiece, simulated with multiple copies in the simulation box, closely resemble the structural properties of the equivalent polypeptides at infinite dilution for both GROMOS force fields used (Fig. 1B, *wild-type_multiple_ and carbonylated_multiple_*). The only appreciable difference concerns the α-helical content in carbonylation simulations where the average value at infinite dilution exceeds that for multiple-copy simulations by approximately 40% (12.1±5.1 vs. 7.0±3.9). Additionally, we observe major unfolding (RMSD>5 Å) for 4 out of 116 simulated wild-type villin headpiece molecules in multiple-copy simulations, which is consistent with the fact that villin headpiece is a marginally stable protein. However, it is noteworthy that two out of these four instances of unfolding occur in simulations obtained using GROMOS54a7 force field in combination with the RF method. Finally, the overall structural stability of wild-type villin headpiece in multiple-copy GROMOS simulations is very similar to that observed in AMBER99SB-ILDN and CHARMM22-CMAP simulations (Table S1).

### Analysis of the aggregation process and villin headpiece aggregates

**Figure 2:**
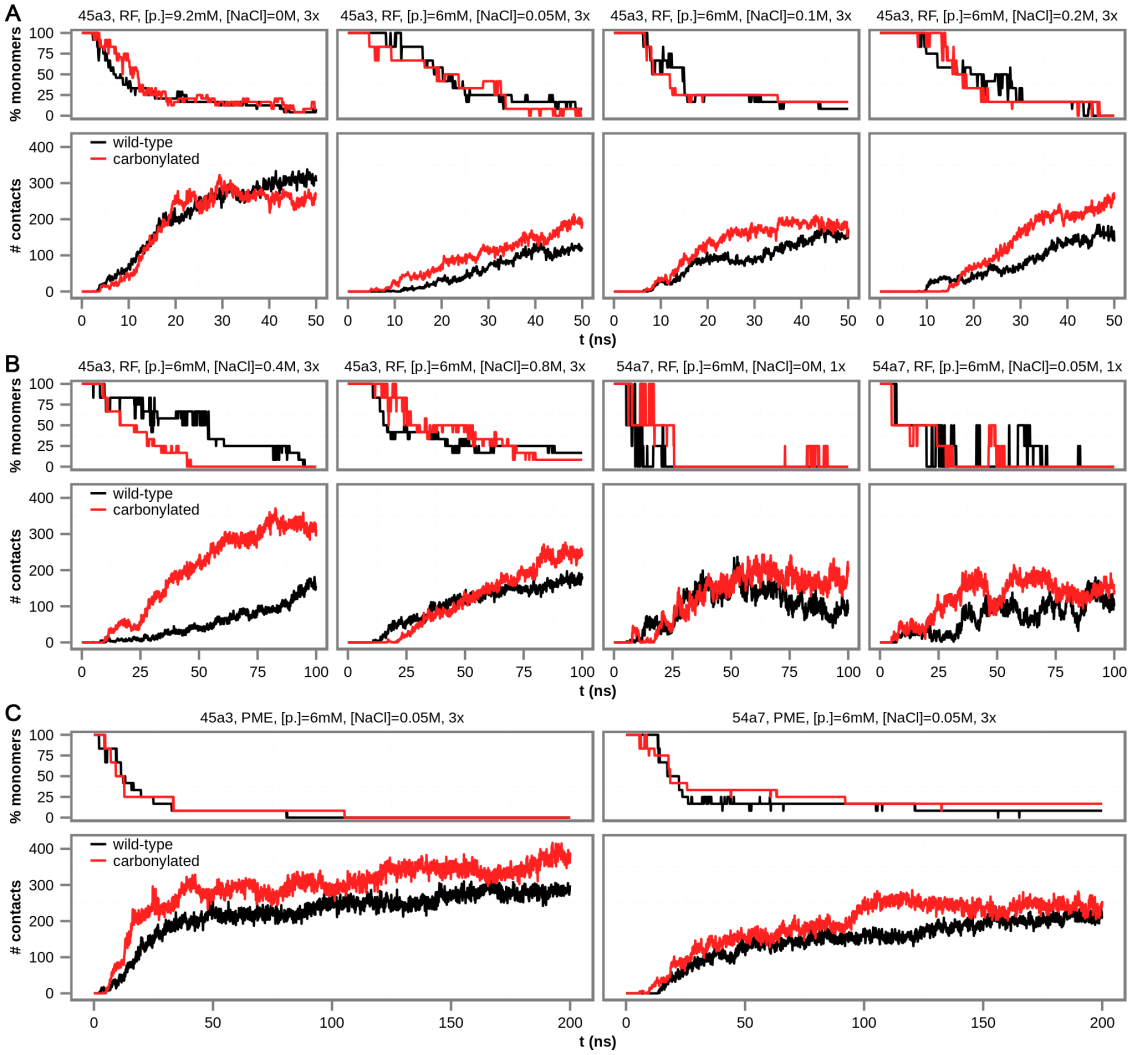
Aggregation of wild-type and carbonylated villin headpiece under various simulation conditions for GROMOS 45a3 and 54a7 force fields. Percentage of free monomers in solution and the number of intermolecular atomic contacts as a function of time, averaged over independent simulations performed under the same conditions. Altogether, 10 simulation setups were examined and are here grouped according to length: A) 50-ns-long, B) 100-ns-long, and C) 200-ns-long simulations. Exact simulation setup is given above each plot including the force field (GROMOS 45a3 or 54a7), type of electrostatics treatment (RF – reaction field, PME – particle mesh Ewald), protein concentration ([p.]), salt concentration ([NaCl]) and the number of replicate trajectories used to generate the curves (1× or 3×).

Altogether, 10 different simulation setups have been examined (Table S1) using GROMOS 45a3 and 54a7 force fields in combination with different types of electrostatics treatment at various ionic strengths. As predicted, carbonylated villin headpiece molecules start to associate after only a few nanoseconds of free diffusion, leading to an exponential decrease in the number of free monomers in solution and a concomitant increase in the number of intermolecular atomic contacts at all conditions examined (Fig. 2, see Methods for more details). Configurations in which the number of free carbonylated monomers reaches 0 are observed in 23 out of 26 simulated trajectories with aggregates comprised of at least 75% of all monomers observed in every single trajectory (Fig. 2). Surprisingly, in contradiction with experimental findings [25], [26], the fraction of free monomers in wild-type villin headpiece simulations decreases over time in the same manner as for the carbonylated villin headpiece and the same is true for the increase in the number of inter-protein atomic contacts for both GROMOS force fields used (Fig. 2). In fact, complete disappearance of free monomers and, at the same time, appearance of aggregates comprised of all simulated monomers, is observed in 22 out of 26 GROMOS trajectories of the wild-type villin headpiece, with aggregation being largely independent of the specific force field or simulation setup used.

To further characterize the aggregation process of the studied systems, we have analyzed the kinetics of protein-protein association and dissociation in simulations obtained using the two GROMOS force fields. The average waiting time for dissociation of individual wild-type molecules from aggregates over all simulated conditions is 45 ns, which is significantly longer than the average waiting time required for their association of 17 ns. Note that the observed association time is in an excellent agreement with the inverse of the diffusion-limited association rate (r_on_^−1^ = (k_on_[villin])^−1^ = 16 ns) as estimated following the formalism of Smoluchowski [44] from the concentration ([villin] = 6 mM), the size and the diffusion coefficient of wild-type villin headpiece monomers directly calculated from simulated trajectories. In this framework, the rate constant is simply given by k_on_ = 4πRD, where R is the sum of radii of interacting molecules (here taken as twice the radius of gyration of villin headpiece) and D is the relative diffusion coefficient. Similarly, the average waiting times of protein dissociation and association for the carbonylated system are 51 ns and 19 ns, respectively. Importantly, approximately 65% of wild-type and 70% of carbonylated complexes that have formed never dissociate during our simulations, including all those with life-times longer than 55 ns (Fig. S1). This fact clearly indicates that the actual life-times of villin headpiece in the aggregated state may be much longer than the average values obtained from simulations, which are limited by sampling. On the other hand, longer simulations would not significantly influence the estimated association times, since only less than 5% of proteins in both systems remain free in solution throughout simulations. This in turn suggests that even much lower protein concentration would likely not prevent wild-type villin headpiece from aggregating, but only increase the search time needed for proteins to find each other by free diffusion.

**Figure 3:**
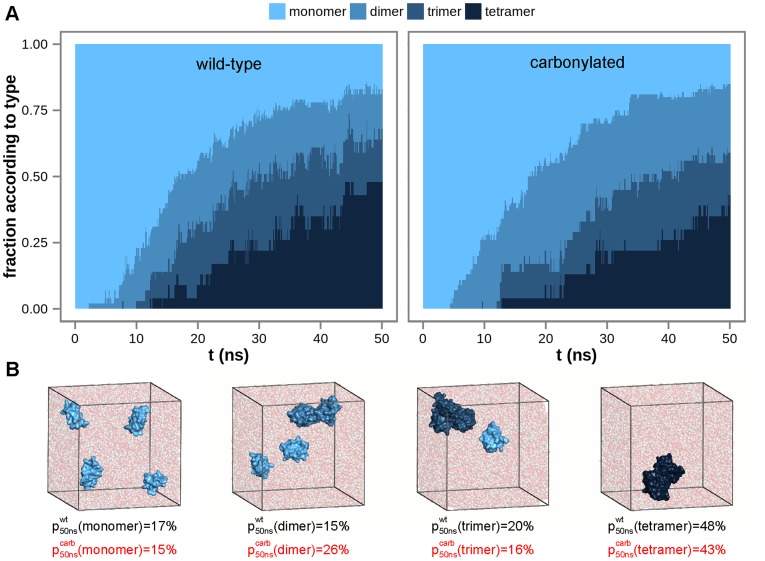
Cumulative degree of wild-type and carbonylated villin headpiece self-association for GROMOS 45a3 and 54a7 force fields. A) average fraction of villin headpiece self-assemblies according to type as a function of time calculated over all GROMOS trajectories. B) snapshots from simulated GROMOS trajectories depicting a typical sequence of formation of villin headpiece aggregates from free monomers. Fraction of monomers, dimers, trimers and tetramers in the ensembles at 50 ns are given explicitly.

**Figure 4:**
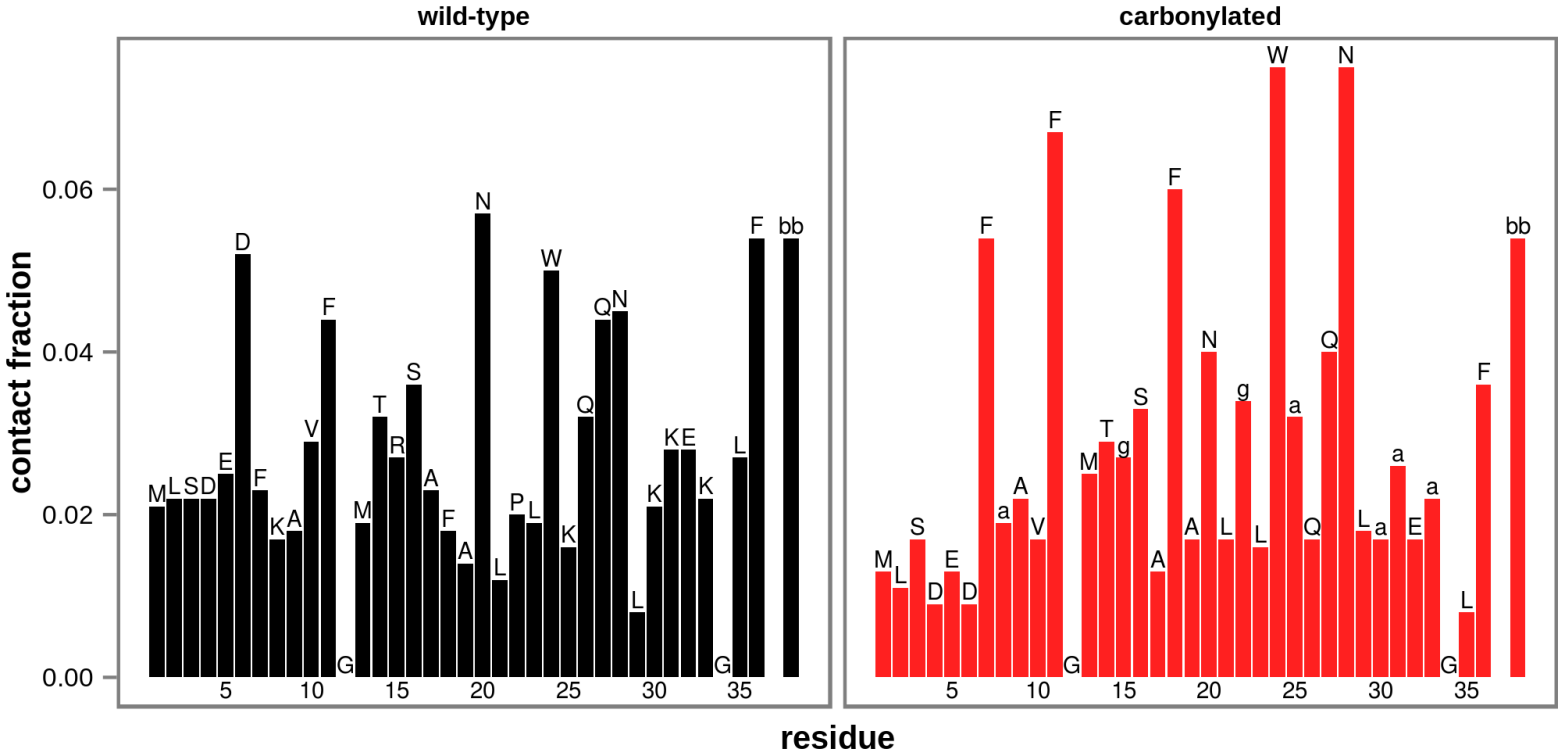
Sequence-wise interaction propensity for GROMOS simulations. Interaction propensity is estimated by the number of intermolecular atomic contacts normalized by solvent-accessibility, i.e., surface-exposure per amino acid (side chains only), with the peptide backbone treated as a separate residue, with the values obtained by averaging over all simulated trajectories (bb – backbone, a – aminoadipic semialdehyde, g – glutamic semialdehyde, while canonical amino acids are indicated using standard 1-letter code).

For both wild-type and carbonylated villin headpiece simulations performed using GROMOS 45a3 and 54a7 force fields, starting from free monomers, peptide dimers begin to form first, followed by the formation of trimers and tetramers, leading to the maximum number of tetrameric aggregates after approximately 50 ns of simulated time (Fig. 3A). Overall, tetrameric aggregates are the most abundant and free monomers the least abundant species for both wild-type and carbonylated villin headpiece simulations, representing on average approximately 45% and 15% of the total protein content at 50 ns in both cases, respectively (Fig. 3B). In order to identify specific residues involved in self-association, we have calculated the fraction of intermolecular atomic contacts formed by each residue (side chains only) over all wild-type and carbonylated GROMOS simulations pooled together separately, including a normalization by solvent-accessibility to account for surface exposure, i.e., probability to interact. Surprisingly, while association of hydrophobic residues is believed to be a key element in protein aggregation, our analysis reveals that interactions in both wild-type and carbonylated aggregates are dominated by either backbone or glutamine and asparagine residues (Fig. 4). In addition to these largely hydrophilic moieties, aggregates are also characterized by contacts involving hydrophobic ring-containing phenylalanines and tryptophans (Fig. 4). Finally, we have also analyzed the average contact maps for both wild-type and carbonylated systems (Fig. S2), which demonstrate that the N-terminal residues form fewer intermolecular contacts than the rest of the molecule in both studied systems. Similarly, the region between residues 15 and 25 of the wild-type contact map is depleted in contacts for the two GROMOS force fields, whereas the carbonylated map displays a somewhat more even distribution of contacts. We should emphasize, however, that no pronounced system-specific pattern among the residues exhibiting a high number of intermolecular contacts has been observed.

### What drives villin headpiece aggregation in MD simulations?

**Figure 5:**
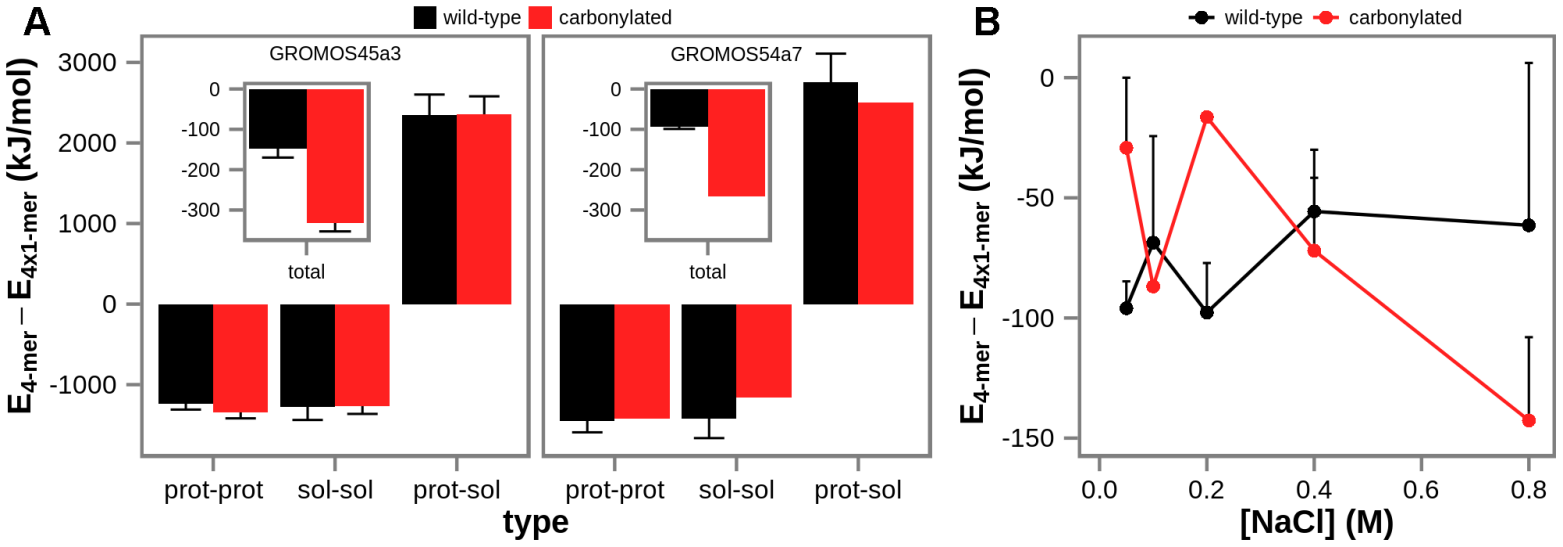
The difference in total potential energy between the fully aggregated (tetrameric) and the non-aggregated (monomeric) conformations of wild-type and carbonylated villin headpiece calculated directly from simulated data. A) average difference in total potential energy (inset, the y-axis the same as in the main figure), and contributions from solvent-solvent, protein-protein and protein-solvent interactions over simulations obtained using PME electrostatics treatment and 0.05 M salt concentration and either GROMOS 45a3 (left panel) or GROMOS 54a7 (right panel) parameter set. B) average difference in total potential energy over simulations obtained using GROMOS 45a3 and RF electrostatics treatment at different salt concentrations. Error bars represent standard deviations from the calculated averages over the independent simulations at the simulation conditions given.

To address this, we have explored the role of enthalpic contributions in the aggregation of the studied systems, including solvent-solvent, protein-solvent and protein-protein interactions, by calculating the average difference in potential energy between the fully aggregated (tetrameric) and the non-aggregated (monomeric) conformers (Fig. 5). Moreover, we have monitored the potential energy as a function of simulated time (Fig. S3). Expectedly, in GROMOS simulations solvent-solvent and protein-protein interactions provide favorable contribution, while protein-solvent interactions provide unfavorable contribution to the total potential energy of aggregation, as seen in the case of simulations with the most extensive sampling (200 ns) and the PME electrostatics treatment (Fig. 5A and S3). However, the total potential energy of the same systems decreases with simulated time (Fig. S3) and is significantly lower in the aggregated state (by approximately 150 kJ/mol and 330 kJ/mol for the wild-type and the carbonylated system simulated by GROMOS45a3 force field, respectively, and approximately 100 kJ/mol and 270 kJ/mol for the wild-type and the carbonylated system simulated by GROMOS54a7 force field, respectively, Fig. 5A), suggesting that self-association may be an enthalpically driven process. Further analysis of the simulations obtained using RF electrostatics treatment (the GROMOS 45a3 parameter set only) shows that the aggregated species are also favored at every salt concentration examined (Fig. 5B).

**Figure 6:**
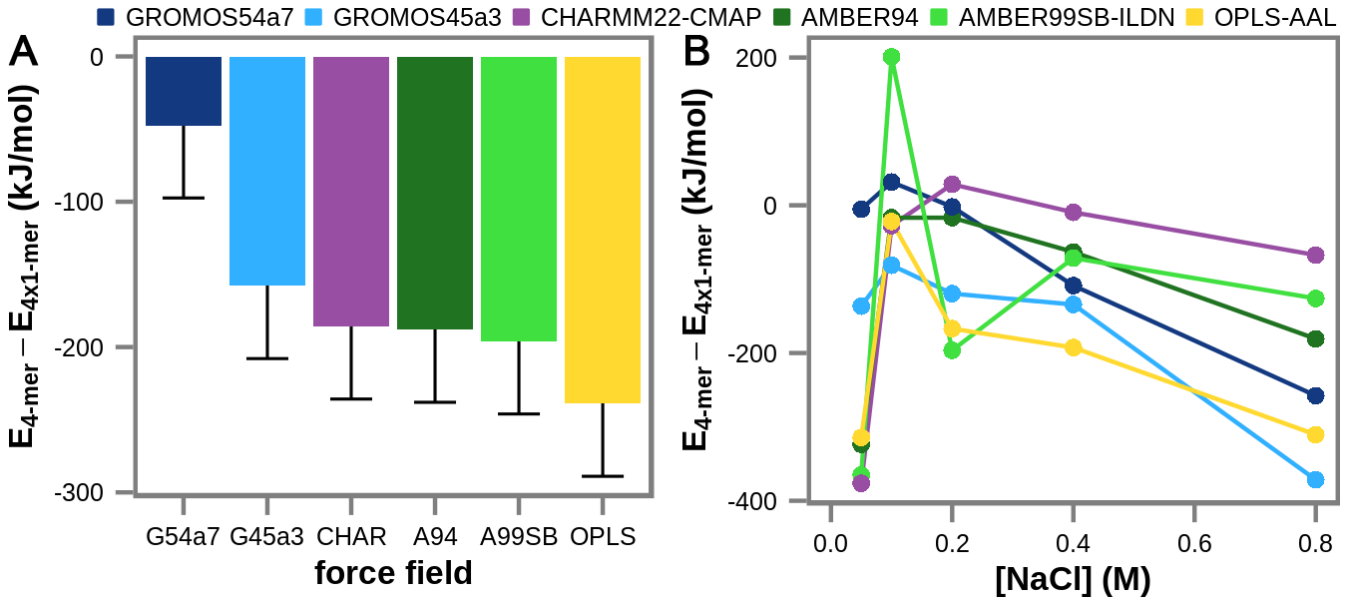
The estimated difference in total potential energy between the fully aggregated (tetrameric) and the non-aggregated (monomeric) conformations of wild-type villin headpiece. The difference is calculated from re-evaluated energies using PME electrostatics treatment and 6 widely used MD force fields on energy-minimized configurations taken from simulation obtained using GROMOS forcer fields. A) averages over all simulations with the estimated standard errors (50 kJ/mol) shown using one-sided error bars. B) averages over simulations at different salt concentrations.

In order to study other force fields in addition to GROMOS 45a3 and 54a7, we have re-evaluated the potential energy of the simulated GROMOS trajectories by employing 4 additional widely used MD force fields (AMBER94, AMBER99SB-ILDN, CHARMM22-CMAP and OPLS-AAL) and two types of electrostatics treatments (RF and PME) on energy-minimized snapshot configurations, where the force field used for energy minimization was also used to re-evaluate the potential energy. For consistency, the same procedure was also repeated for the GROMOS 45a3 and 54a7 force fields as well. Note that we have only re-evaluated potential energies for wild-type systems since parameters for carbonylated residues are only available for the GROMOS force fields. Moreover, for all force fields other than the two GROMOS force fields, we have used the TIP3P water model [43] in the above procedure. Remarkably, regardless of the type of electrostatics treatment, all of the evaluated force fields show the same trend in favoring the tetrameric aggregated over the monomeric non-aggregated state of wild-type villin headpiece (Fig. 6A and S4) with the extent of such bias ranging from approximately −50 kJ/mol to −240 kJ/mol (PME calculations) for GROMOS54a7 and OPLS-AAL force fields, respectively. Importantly, in order to decrease the uncertainty in estimation, we have averaged the re-evaluated differences in potential energy between the tetrameric and monomeric configurations for each force field over all trajectories no matter which simulation condition they came from (Fig. 6A and S4A). Under the assumption that the entropic contribution to the free energy of association is comparable for all different force fields, this result suggests that utilizing any of these force fields would most likely lead to aggregation of wild-type villin headpiece in MD simulations, and therefore be at odds with experimental observations. Moreover, while different force fields exhibit different dependence on salt when it comes to aggregation, they all favor the aggregated state at most conditions examined (Fig. 6B). Interestingly, the difference in potential energy of tetrameric and monomeric configurations reaches a maximum at either 0.2 M NaCl (CHARMM22-CMAP) or 0.1 M NaCl (all other force fields) (Fig. 6B). The analysis of this effect, which is possibly related to the different chaotropic behavior of NaCl at different concentrations, is nevertheless beyond the scope of our present study. Finally, we should emphasize that our energy re-evaluation procedure is only approximate: the calculated differences in the total potential energy come with error bars of approximately 200 kJ/mol for each simulation and 50 kJ/mol for the average difference (Figs. 6 and S4), as estimated using the root-mean-square errors from the potential energies derived directly from simulations, calculated for the same GROMOS force fields as used in the simulations.

**Figure 7:**
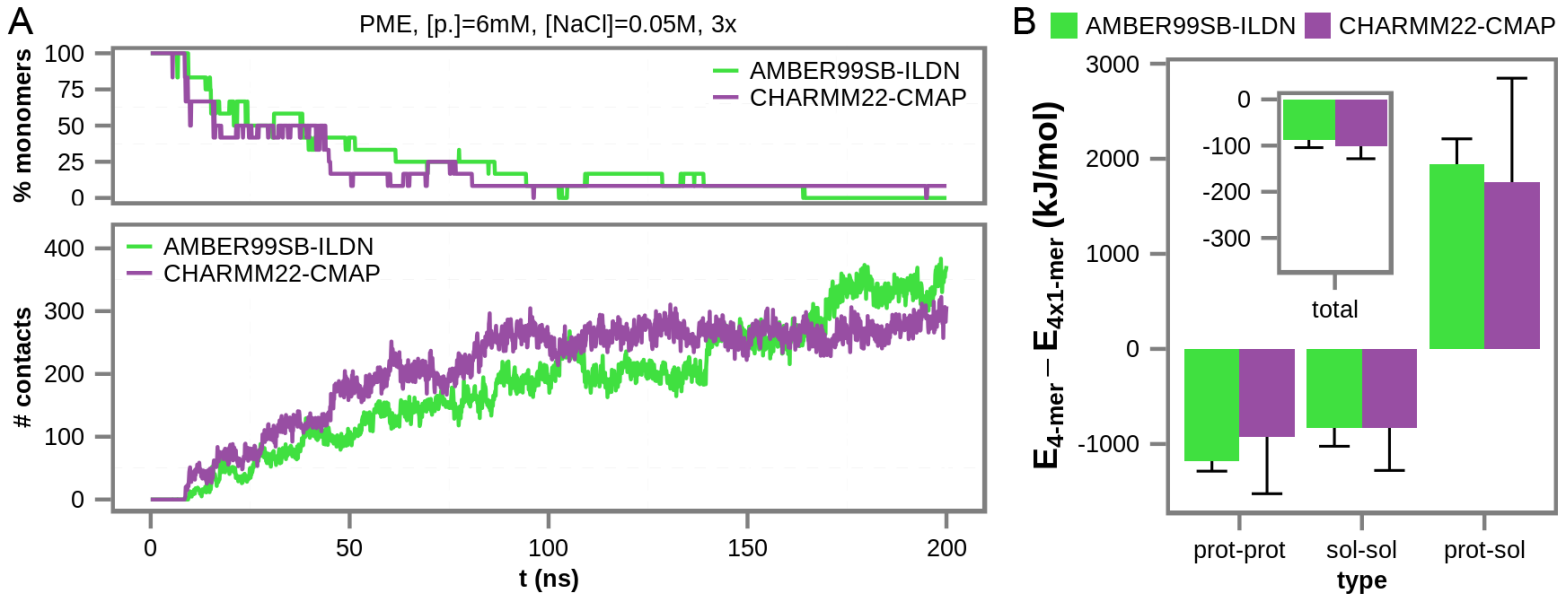
Aggregation of wild-type villin headpiece simulated using AMBER99SB-ILDN and CHARMM22-CMAP force fields. A) the percentage of free monomers in solution and the number of intermolecular atomic contacts as a function of time, averaged over three independent simulations performed using a given force field. The type of electrostatics treatment (PME – particle mesh Ewald), protein concentration ([p.]), salt concentration ([NaCl]) and the number of replicate trajectories used to generate the curves (3×) are given above the graph. B) the average difference in potential energy between the fully aggregated (tetrameric) and the non-aggregated (monomeric) state in total potential energy (inset, the y-axis the same as in the main figure), and contributions from solvent-solvent, protein-protein and protein-solvent interactions over simulations obtained using a given force field. Error bars represent standard deviations from the calculated averages over the independent simulations obtained using AMBER99SB-ILDN and CHARMM22-CMAP force fields.

In order to further explore the above findings, we have additionally simulated the wild-type villin headpiece using AMBER99SB-ILDN [40] and CHARMM22-CMAP [41] force fields, focusing on simulation conditions ([protein] = 6 mM and [NaCl] = 0.05 M), which are most similar to the experiment described in reference [25]. Note that carbonylated versions of the peptide were not modeled with these force fields since parameters for carbonylated amino acids were not currently available. Similarly to trajectories obtained using GROMOS force fields, for both AMBER99SB-ILDN and CHARMM22-CMAP force fields, the number of intermolecular atomic contacts increases and the number of free monomers decreases with concomitant decrease in the potential energy of the system over time, as shown by the averages over three 200 ns-long independent simulations (Fig. 7A and Fig. S5). Indeed, for the AMBER99SB-ILDN force field, the long-time fraction of free monomers drops down to 0, while for CHARMM22-CMAP it stabilizes at a low 8.3%. Importantly, the difference in total potential energy between the aggregated tetrameric and the non-aggregated monomeric state for both force fields is approximately −100 kJ/mol (Fig. 7B), which is similar to the value observed in simulations generated using GROMOS54a7 force field and PME electrostatics treatment (Fig. 5A). On the other hand, these numbers are by almost 100 kJ/mol less negative than the values obtained by re-evaluating the energies of the snapshots obtained in GROMOS simulations under similar conditions (Fig. 6A and 7B). This further suggests that, although qualitatively likely correct, our energy re-evaluation procedure, especially in the case of individual salt conditions (Fig. 6B), is quantitatively only approximate as already discussed above.

## Discussion

By probing various simulation conditions, we have observed that simulated wild-type and carbonylated villin headpiece molecules aggregate in a similar fashion, despite the fact that they display markedly different aggregation propensities [32]. This finding is even more remarkable given the fact that we have focused on conditions experimentally known to render the wild-type villin headpiece soluble. Villin headpiece self-association in our simulations is accompanied by a decrease in protein-protein and solvent-solvent, and an increase in protein-solvent short-range potential energy, resulting in a significant net decrease of total potential energy upon aggregation. Strikingly, by re-evaluating the potential energy of the aggregated and non-aggregated conformers from GROMOS simulations using 6 widely-used atomistic force fields, we have shown that all of them favor the aggregated state (Fig. 6 and S4). Here, one should emphasize that this result was obtained by averaging over conformers originally obtained by differently defined Hamiltonians and using a limited number of energy minimized configurations, significantly increasing the error in the estimated potential energy (see Methods for more details). These shortcomings notwithstanding, the general trends for all force fields consistently point in the direction of favoring aggregation. Moreover, this result was further confirmed by direct simulations of villin headpiece at high concentration using AMBER99SB-ILDN and CHARMM22-CMAP force fields (Fig. 7 and S5), in which spurious aggregation was also observed, albeit for a single simulation setup only. While more extensive simulation efforts are undeniably needed to complete the analysis of different atomistic force fields, we believe that our present results do reveal significant deficiencies in how they capture the behavior of proteins in crowded solutions and invite caution when it comes to using them for such purposes.

Even though it is experimentally known that villin headpiece can begin to aggregate at protein concentration of 6 mM after a period of a few days [25], it is highly unlikely that this is related to the nanosecond-time-scale aggregation observed here. In order to address the possibility that aggregation of the wild-type system was still induced by the “borderline” villin headpiece concentrations used, we have analyzed the kinetics of protein-protein association and dissociation. This analysis has revealed that a large number of protein-protein complexes never dissociate in the course of simulated trajectories, and that the average protein dissociation time is markedly longer than the average association time (Fig. S1), suggesting that aggregation in MD simulations would occur even at lower concentrations than applied here. Having said this, it is important to emphasize that in our analysis we do not directly compare simulated and experimental observables reporting on villin headpiece aggregation, but rather rely on indirect interpretation of different experimental observables. It is well known that such comparisons at the level of interpretation can be fraught with difficulties and are never fully unambiguous [45], [46]. For example, it is possible that the CD spectra of tetrameric aggregates are similar to those of the monomeric form and that the experimental interpretation of such spectra, which was used to suggest that villin headpiece does not aggregate at the concentration of 9.2 mM, was simply inaccurate [24]. On the other hand, both 2D IR and NMR are extremely sensitive to local changes in the environment of individual polypeptide groups and have successfully been used to detect dimerization, multimerization and aggregation of different proteins [47]–[52]. This further suggests that villin headpiece is indeed monomeric at concentrations studied herein, as determined in the experiments performed by using these methods [25]–[28]. In conjunction with our analysis of residence times in different states, this in turn bolsters the claim that the aggregation observed in our simulations is indeed unphysical, but the above caveats should still be borne in mind.

Our analysis shows that interactions in villin headpiece aggregates are mainly dominated by polar glutamines and asparagines as well as hydrophobic ring-containing phenylalanines and tryptophans (Fig. 4). Although the two former hydrophilic amide-containing amino acids are generally considered to be aggregation reducing [33], [53], they have been repeatedly linked to protein aggregation and deposition disorders, most notably in poly-Q diseases [5], [20], [54]. In addition to this, the high occurrence of peptide backbone atoms among villin headpiece intermolecular contacts (Fig. 4) supports the hypothesis that poly-peptide chains have a general tendency to aggregate due to intrinsic aggregability of the protein backbone [55]. This is further corroborated by recent findings that poly-glycine and poly-alanine chains aggregate readily [56]. Finally, Andrews and Elcock have recently used MD simulations in combination with several atomistic force fields and water models to analyze the behavior of high-concentration aqueous solutions of glycine, valine, phenylalanine and asparagine [16]. In agreement with the available experimental data, their analysis shows that asparagine and phenylalanine exhibit a considerably greater density increment, a measure of interaction propensity, when compared to glycine and valine, which is also well matched by our present data.

Taken together, our results suggest that typical classical MD force fields possibly bias protein aggregation by overestimating protein-protein and solvent-solvent as opposed to protein-solvent interactions. Parenthetically, the primary strategy in parameterizing the GROMOS 54a7 force field, which exhibits one of the lowest preference towards the aggregated state when compared to other evaluated force fields, was to reproduce experimental hydration free energies of amino-acid side-chain analogs, a property closely related to solubility in water, while the other force fields examined here significantly underestimate this property [57]–[59]. Overall, the imbalance between protein-protein, protein-solvent and solvent-solvent components of total potential energy may be partly a consequence of the widely-used force field validation approaches, which frequently aim at reproducing secondary and tertiary structures of well-characterized proteins. Simply put, strengthening of protein-protein and weakening of protein-solvent interactions leads to stabilization of protein structure and this can have direct repercussions on the behavior of proteins in crowded environments, as shown here. This, if true, further suggests that realistic polypeptides may display more dynamics and unstructuredness than generally observed in MD-simulation studies. Interestingly, a recent study exploring the limitations of MD simulations by employing CHARMM22* force field [60] and a state-of-the-art designated supercomputer to perform a 200-µs-long simulation of an intrinsically unfolded protein revealed that the modeled protein appears to be more compact and collapsed than observed by NMR [61], further supporting this speculation. Importantly, these potential flaws of current force fields may have strong implications when it comes to the accuracy of MD models in describing protein dynamics and interactions in biologically relevant crowded environment. Recent validation studies of force fields show that they improve over time, but are still not able to reproduce all relevant experimental data [62], [63]. Such synergistic efforts between experiment and MD simulations should lead to improvements in computational models of biomolecular systems in the context of experimentally or biologically realistic conditions. We hope that the results presented herein will provide a new source of motivation in this direction.

## Methods

### Molecular dynamics simulations setup

Classical MD simulations were used to study the behavior of villin headpiece with multiple copies of the molecule in the simulation box. We examined both wild-type villin headpiece (sequence: MLSDEDF**K**AVFGMT**R**SAFANL**P**LW**K**QQNL**KK**E**K**GLF) and its carbonylated form. The seven letters in bold mark the most important carbonylable amino acids (K, R and P) in villin headpiece, which were all modified in the carbonylated form of the molecule. Upon carbonylation, lysine is converted into aminoadipic-semialdehyde, while arginine and proline are converted into glutamic-semialdehyde, for which force field parameters were taken from refs [32], [64], [65]. Ten simulation protocols were applied (Table S1), varying in protein (6 mM and 9.2 mM) and salt (0 M, 0.05 M, 0.1 M, 0.2 M, 0.4 M and 0.8 M) concentration, force field (GROMOS 45a3 and 54a7 parameter sets), electrostatics treatment (RF - reaction-field [38] and PME - particle mesh Ewald [37]), the number of protein molecules in a simulated box (4 and 8), simulation time (50 ns, 100 ns and 200 ns), and the number of independent simulations (1 and 3). Additionally, the wild-type villin headpiece was simulated at 6 mM protein concentration and 0.05 M salt concentration using AMBER99SB-ILDN [40] and CHARMM22-CMAP [41] force fields, PME electrostatic treatment and three 200 ns-long independent replicas in each case (Table S1). All MD simulations were carried out using GROMACS biomolecular simulation package [66], keeping the system at 300 K and 1 bar using a Berendsen thermostat (τ*_T_* = 0.05 ps) and barostat (τ*_p_* = 1 ps and compressibility = 4.5×10^−5^ bar^−1^) [67]. A cutoff of 1.4 nm for both Lennard-Jones and Coulomb potentials was used with the dielectric constant of 65 for RF, and the Fourier spacing of 0.1 nm for PME calculations. Starting from either wild-type or carbonylated free monomers (4 and 8 for 6 mM and 9.2 mM protein concentration, respectively) maximally separated and randomly oriented in a simulation box, polypeptides were allowed to diffuse freely and interact with each other and the solvent. The initial configurations were prepared by solvating villin headpiece molecules in a cubic simulation box, with the size defined by the protein concentration, i.e., sides of approximately 10.4 nm (6 mM) and 11.3 nm (9.2 mM), for a total number of atoms exceeding 100,000 in all cases (see Table S1 for more details). Villin headpiece monomers were randomly selected from the ensemble of the last 25 ns of five 110-ns-long independent simulated trajectories of both the peptide in the wild-type and carbonylated form from ref [32], only taking into account structures with atom-positional backbone root-mean-square deviation (RMSD) from the native villin headpiece structure [23] in the range of the average ensemble plus or minus one standard deviation. After filling the simulation box with SPC water molecules [36] for GROMOS 45a3 and 54a7 simulations or TIP3P water molecules [43] for AMBER99SB-ILDN and CHARMM22-CMAP simulations, sodium chloride was added at a given concentration by replacing the equivalent number of randomly chosen water molecules, all the while ensuring charge neutrality with an excess of 2 Cl^−^ and 4 Na^+^ ions per villin headpiece molecule in the case of wild-type and carbonylated systems, respectively. Following steepest descent energy minimization (1500 steps), the system was equilibrated by gradually increasing the temperature (from 100 to 300 K) over 100 ps with gradually decreasing position restraints (from 25000 to 5000 kJ mol^−1^ nm^−2^) at constant volume and temperature, and finally additionally equilibrated for 20 ps at constant pressure and temperature of 1 bar and 300 K. Atom coordinates and velocities were saved every 50,000 integration steps, i.e. 100 ps. The total simulated time for all setups exceeds 6.4 µs.

### Potential energy re-evaluation

To examine the performance of 6 widely used MD force fields (GROMOS45a3 [34], GROMOS54a7 [35] AMBER94 [39], AMBER99SB-ILDN [40], CHARMM22-CMAP [41] and OPLS-AAL [42]) in the context of villin headpiece aggregation, the trajectories of the wild-type villin headpiece simulated using GROMOS 45a3 and 54a7 force fields were re-evaluated as follows: first, utilizing a given force field, each saved configuration was energy minimized by steepest descent in 1500 steps, and second, the total potential energy was calculated for the minimized configurations using the same force field as in the energy minimization step, together with contributions from short-range non-bonded solvent-solvent, protein-protein and protein-solvent interactions, which were evaluated for all atom pairs within a cutoff distance of 1.4 nm. Electrostatic contribution to the potential energy was calculated using RF and PME methods with the same parameters as used in the simulation setups. The water TIP3P [43] model was used to re-evaluate potential energies of AMBER94 [39], AMBER99SB-ILDN [40], CHARMM22-CMAP [41] and OPLS-AAL [42] force fields.

### Analysis of the simulated data and figure preparation

The collected trajectories were analyzed primarily using GROMACS tools [66], except for secondary structure analysis, where DSSP [68] was used. PYMOL [69] and ggplot2 [70] (from R) tools were used to generate the figures. Backbone atoms were used for roto-translational fitting and calculation of atom-positional root-mean-square deviation (RMSD) with respect to the NMR villin headpiece structure (first model) [23]. The number of inter-peptide atomic contacts and villin headpiece association and dissociation kinetics were evaluated by defining atomic contacts to be present any time two heavy atoms or hydrogen atoms bound to them from different villin headpiece molecules come within 0.4 nm from each other, and a complex to be present any time a pair of villin headpiece molecules remain continuously in atomic contact in the course of at least 1 ns. The site-specific intermolecular interaction propensity along the villin headpiece sequence was estimated by calculating the fraction of the number of intermolecular atomic contacts per residue (normalized at each time-point by the solvent-accessibility of a residue in question) in each simulation, and subsequently averaged over all simulated trajectories (either wild-type or carbonylated). The solvent-accessibility, i.e. the surface-exposure of a given residue was calculated as the fraction of the solvent-accessible surface area (SASA) of the residue in the total SASA of the protein. Note that only amino-acid side chains were used for this whole analysis, while the backbone atoms were considered as a separate collective group. Finally, the differences in potential energy between aggregated and non-aggregated villin headpiece conformers were calculated as the difference between the average potential energy of aggregated (all-tetramer) snapshots and the average potential energy of non-aggregated (free monomers) snapshots from each simulated trajectory, averaged over different simulation sets. In particular, energy differences evaluated directly from simulations were averaged over the independent simulations of each simulation protocol, whereas those calculated from re-evaluated energies were averaged over all simulated systems using GROMOS force fields (excluding simulations at 9.2 mM protein concentration for these cases). Only simulated trajectories with more than 20 snapshots of both aggregated and non-aggregated states were taken into account. Note that villin headpiece systems with protein concentration of 9.2 mM (1 out of 10 simulated setups) were excluded from calculation of average properties if the property in question was either extensive or concentration dependent, including evaluations of villin headpiece association/dissociation kinetics, time-dependent formation of higher-order villin headpiece complexes (e.g. dimers) and potential energy.

## Supporting Information

Figure S1 **Dissociation kinetics of villin complexes in wild-type and carbonylated forms for the GROMOS force fields.** Main panel - the percentage of protein-protein complexes that never dissociate in the course of simulated trajectories shown as a function of the life-time of the complex. Inset – inverse of the cumulative distribution of the number of villin headpiece intermolecular complexes as a function of the life-time of the complex, i.e., the function that at each value of τ (complex life-time) gives the number of complexes with a longer life-times than the given τ-value, shown for complexes that dissociate (left) and never dissociate (right) over simulated time.
(TIF)

Figure S2 **Sequence-wise contact map of intermolecular atomic contacts in GROMOS simulations of wild-type and carbonylated villin headpiece.** Interaction propensities are estimated by the number of intermolecular atomic contacts normalized by solvent-accessibility, i.e., surface-exposure per amino acid. The color code of the heat map corresponds to the negative logarithm of the number of contacts for each pair of residues, additionally rescaled in such a way that 0 represents the pair of residues with the smallest number of contacts.
(TIF)

Figure S3 **Total potential energy and contributions from solvent-solvent, protein-protein and protein-solvent interactions, shown as a function of time for GROMOS 45a3 and 54a7 simulations.** Curves are shifted to 0 kJ/mol at the initial time point and are calculated as the average over three independent simulations obtained: 1) using the GROMOS 45a3 parameter set, PME electrostatics treatment and 0.05 M salt concentration (left), and 2) using the GROMOS 54a7 parameter set, PME electrostatics treatment and 0.05 M salt concentration (right) for the wild-type (top) and carbonylated (bottom) systems.
(TIF)

Figure S4 **The difference in total potential energy between the fully aggregated (tetrameric) and the non-aggregated (monomeric) state of wild-type villin headpiece.** The difference is calculated from the re-evaluated energies using RF electrostatics treatment and 6 widely used MD force fields on energy-minimized configurations, where the force field used for re-evaluation of the potential energy was also used for energy-minimization. A) averages over all simulations with the estimated standard errors (50 kJ/mol) shown using one-sided error bars. B) averages over simulations at different salt concentrations.
(TIF)

Figure S5 **Total potential energy and contributions from solvent-solvent, protein-protein and protein-solvent interactions, shown as a function of time for AMBER99SB-ILDN and CHARMM22-CMAP force fields.** Curves are shifted to 0 kJ/mol at the initial time point and are calculated as the average over three independent simulations obtained using AMBER99SB-ILDN (left) and CHARMM22-CMAP (right) force fields.
(TIF)

Table S1 **Simulation conditions and setups.** The averages and standard deviations of three measures of foldedness are shown: atom-positional backbone root-mean-square deviation (RMSD) from the native villin headpiece structure, number of residues in α-helical conformation (#α = 19 for native experimental structure) and the sum of distances between the centers of mass of core phenylalanines (ΣdPHEs = 18.0 Å for native experimental structure [23]). The last two rows are the aggregate averages over all 10 simulated systems with multiple copies of the protein (GROMOS force fields only) and the averages over the infinitely diluted systems (data taken from ref [32]), together with their respective standard deviations. The averages for the systems with multiple copies of the protein were calculated over the entire simulated time, whereas for the infinitely diluted systems the last 25 ns of the simulated trajectories were used. The averages and standard deviations are shown for wild-type (left) and carbonylated (right) systems.
(DOCX)
