# Supplementary figures and images for: Are Current Atomistic Force Fields Accurate Enough to Study Proteins in Crowded Environments?
Source: PLoS Comput Biol. 2014 May 22;10(5):e1003638. doi: 10.1371/journal.pcbi.1003638 (PMC4031056; doi:10.1371/journal.pcbi.1003638)

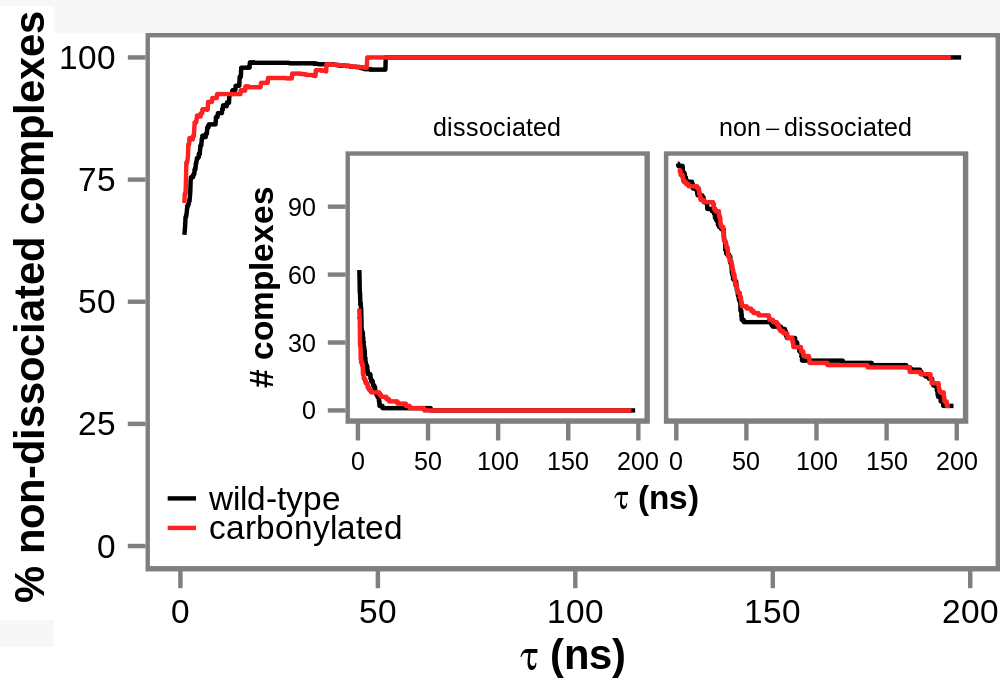

Supplement: Figure S1 — Dissociation kinetics of villin complexes in wild-type and carbonylated forms for the GROMOS force fields. Main panel - the percentage of protein-protein complexes that never dissociate in the course of simulated trajectories shown as a function of the life-time of the complex. Inset – inverse of the cumulative distribution of the number of villin headpiece intermolecular complexes as a function of the life-time of the complex, i.e., the function that at each value of τ (complex life-time) gives the number of complexes with a longer life-times than the given τ-value, shown for complexes that dissociate (left) and never dissociate (right) over simulated time. (TIF) [file pcbi.1003638.s001.tif]

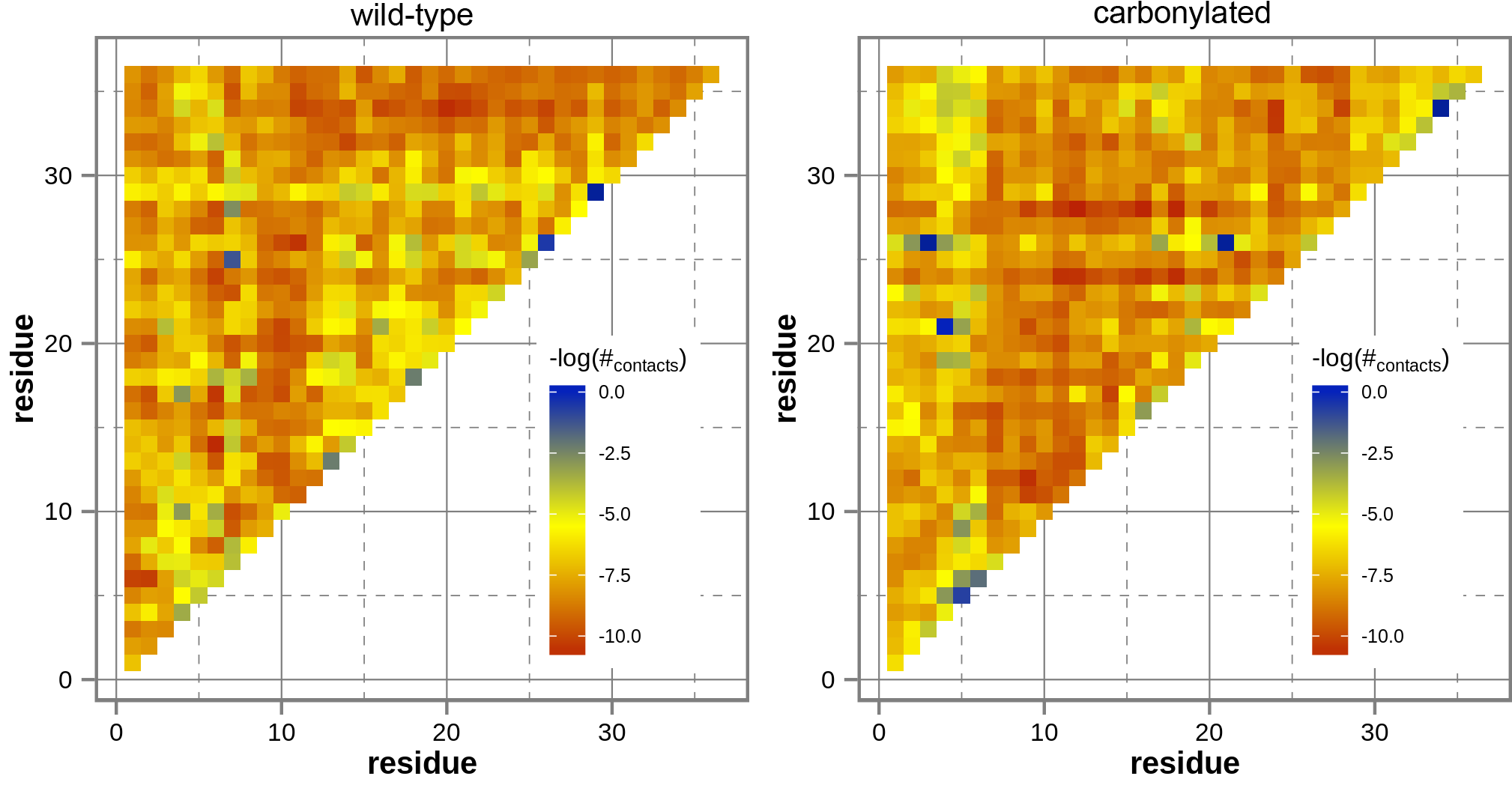

Supplement: Figure S2 — Sequence-wise contact map of intermolecular atomic contacts in GROMOS simulations of wild-type and carbonylated villin headpiece. Interaction propensities are estimated by the number of intermolecular atomic contacts normalized by solvent-accessibility, i.e., surface-exposure per amino acid. The color code of the heat map corresponds to the negative logarithm of the number of contacts for each pair of residues, additionally rescaled in such a way that 0 represents the pair of residues with the smallest number of contacts. (TIF) [file pcbi.1003638.s002.tif]

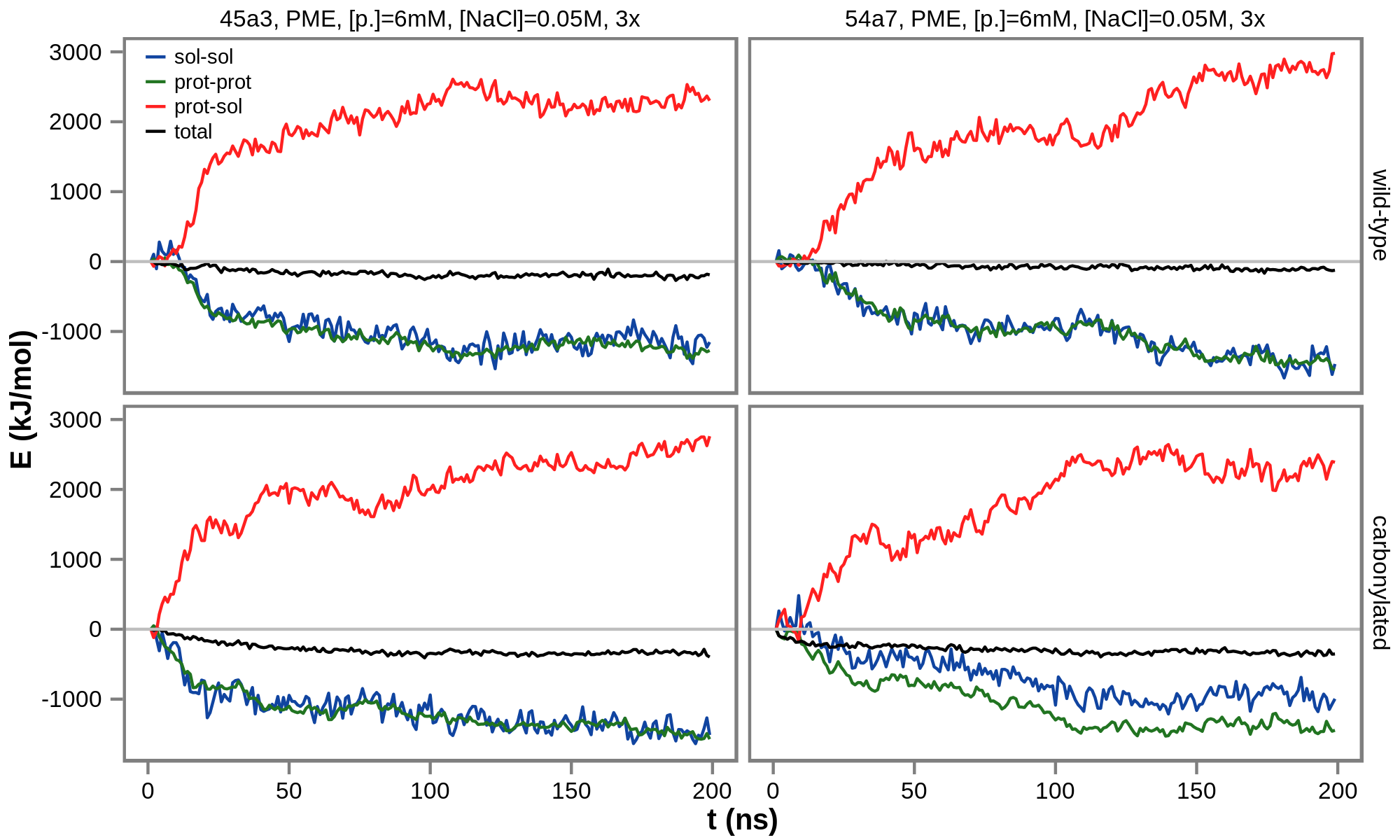

Supplement: Figure S3 — Total potential energy and contributions from solvent-solvent, protein-protein and protein-solvent interactions, shown as a function of time for GROMOS 45a3 and 54a7 simulations. Curves are shifted to 0 kJ/mol at the initial time point and are calculated as the average over three independent simulations obtained: 1) using the GROMOS 45a3 parameter set, PME electrostatics treatment and 0.05 M salt concentration (left), and 2) using the GROMOS 54a7 parameter set, PME electrostatics treatment and 0.05 M salt concentration (right) for the wild-type (top) and carbonylated (bottom) systems. (TIF) [file pcbi.1003638.s003.tif]

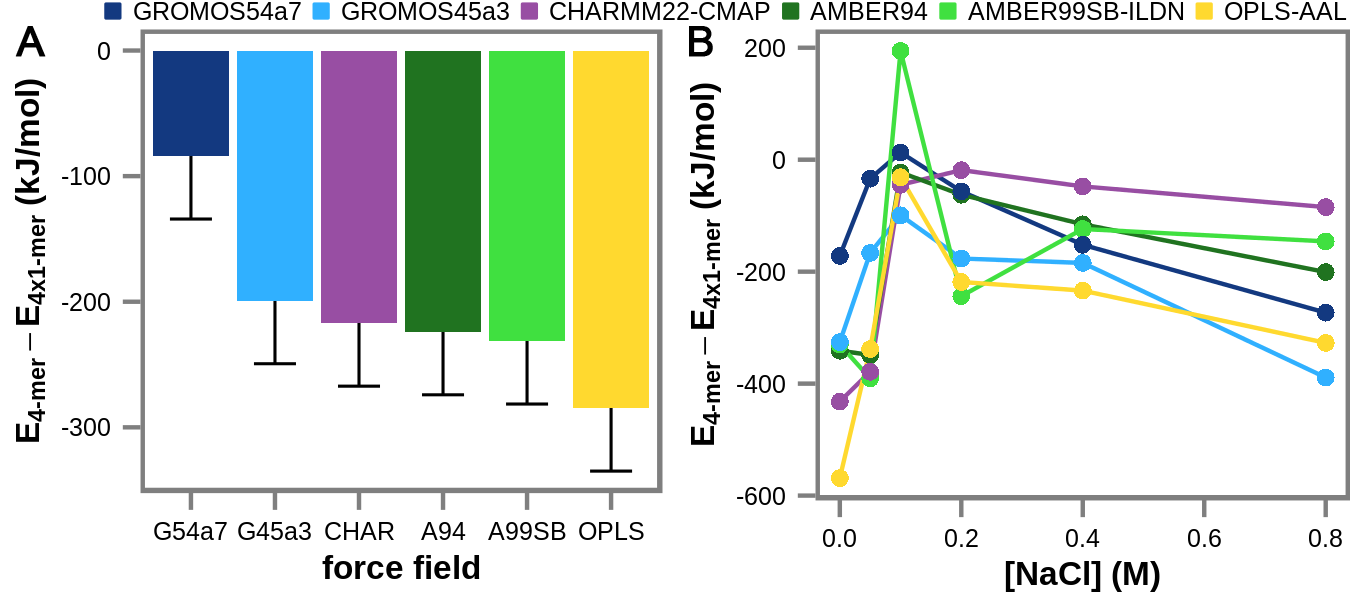

Supplement: Figure S4 — The difference in total potential energy between the fully aggregated (tetrameric) and the non-aggregated (monomeric) state of wild-type villin headpiece. The difference is calculated from the re-evaluated energies using RF electrostatics treatment and 6 widely used MD force fields on energy-minimized configurations, where the force field used for re-evaluation of the potential energy was also used for energy-minimization. A) averages over all simulations with the estimated standard errors (50 kJ/mol) shown using one-sided error bars. B) averages over simulations at different salt concentrations. (TIF) [file pcbi.1003638.s004.tif]

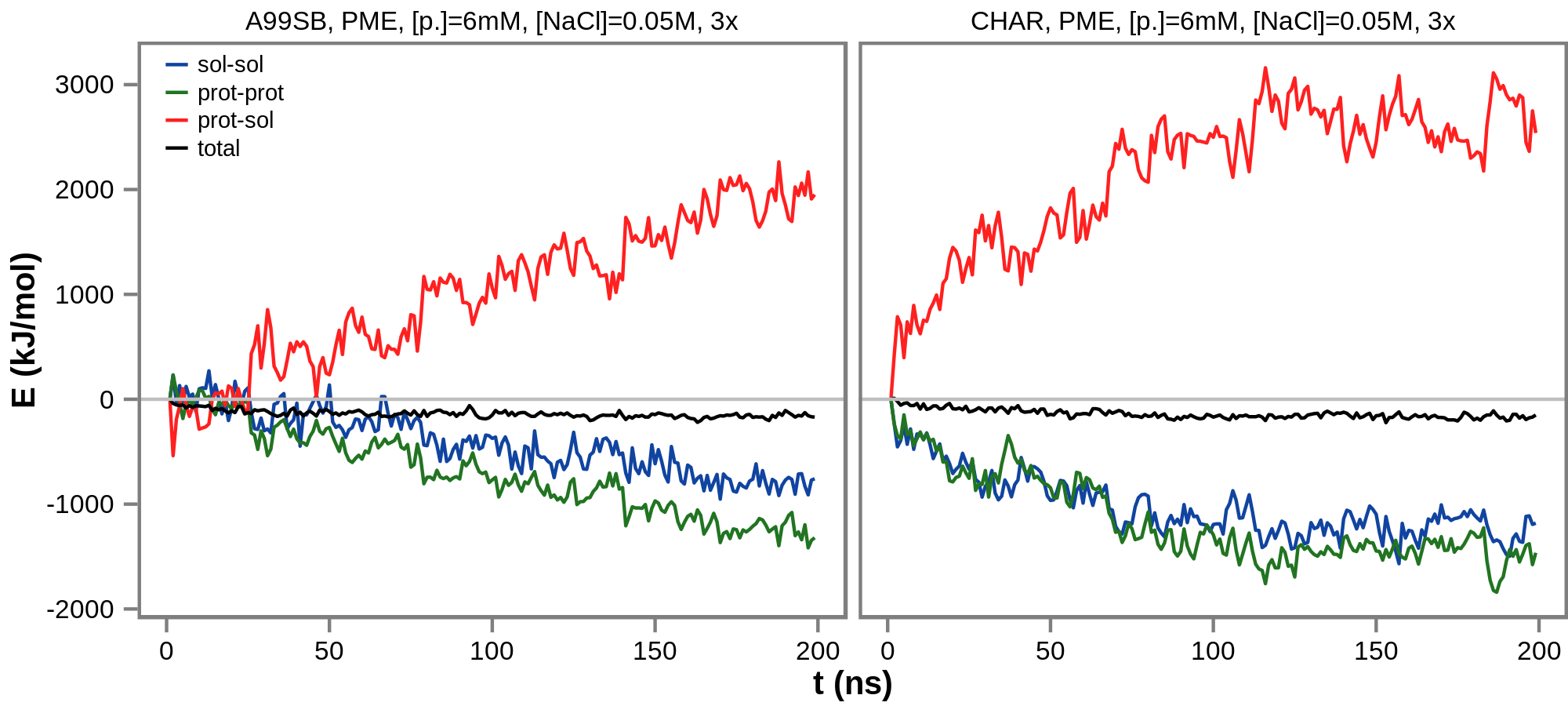

Supplement: Figure S5 — Total potential energy and contributions from solvent-solvent, protein-protein and protein-solvent interactions, shown as a function of time for AMBER99SB-ILDN and CHARMM22-CMAP force fields. Curves are shifted to 0 kJ/mol at the initial time point and are calculated as the average over three independent simulations obtained using AMBER99SB-ILDN (left) and CHARMM22-CMAP (right) force fields. (TIF) [file pcbi.1003638.s005.tif]
