# Supplementary material for: Are Current Atomistic Force Fields Accurate Enough to Study Proteins in Crowded Environments?
Source: PLoS Comput Biol. 2014 May 22;10(5):e1003638. doi: 10.1371/journal.pcbi.1003638 (PMC4031056; doi:10.1371/journal.pcbi.1003638)
Supplement: Table S1 — Simulation conditions and setups. The averages and standard deviations of three measures of foldedness are shown: atom-positional backbone root-mean-square deviation (RMSD) from the native villin headpiece structure, number of residues in α-helical conformation (#α = 19 for native experimental structure) and the sum of distances between the centers of mass of core phenylalanines (ΣdPHEs = 18.0 Å for native experimental structure [23]). The last two rows are the aggregate averages over all 10 simulated systems with multiple copies of the protein (GROMOS force fields only) and the averages over the infinitely diluted systems (data taken from ref [32]), together with their respective standard deviations. The averages for the systems with multiple copies of the protein were calculated over the entire simulated time, whereas for the infinitely diluted systems the last 25 ns of the simulated trajectories were used. The averages and standard deviations are shown for wild-type (left) and carbonylated (right) systems. (DOCX) [file pcbi.1003638.s006.docx]

**Table S1**

| **#** | **[protein] (mM)** | **N peptides** | **[salt] (M)** | **force field** | **electrostatics** | **t (ns)** | **# atoms** | **N simulations** | **RMSD (Å)** | **# alpha** | **dPHE (Å)** |
| --- | --- | --- | --- | --- | --- | --- | --- | --- | --- | --- | --- |
|  |  |  |  |  |  |  |  |  | (wt. \| car.) | (wt. \| car.) | (wt. \| car.) |
| **1** | 9.2 | 8 | 0 | GROMOS45a3 | RF | 50 | ∼140,000 | 3 | 2.7 ± 1.1 \| 8.2 ± 0.8 | 22.6 ± 1.9 \| 6.3 ± 3.8 | 19.9 ± 1.7 \| 34.9 ± 2.3 |
| **2** | 6 | 4 | 0.05 | GROMOS45a3 | RF | 50 | ∼110,000 | 3 | 2.4 ± 0.8 \| 8.5 ± 0.7 | 22.9 ± 1.5 \| 5.3 ± 2.0 | 20.3 ± 1.7 \| 38.2 ± 3.4 |
| **3** | 6 | 4 | 0.1 | GROMOS45a3 | RF | 50 | ∼110,000 | 3 | 2.8 ± 1.8 \| 8.4 ± 0.6 | 22.6 ± 1.9 \| 6.2 ± 3.2 | 19.5 ± 1.0 \| 37.5 ± 3.6 |
| **4** | 6 | 4 | 0.2 | GROMOS45a3 | RF | 50 | ∼110,000 | 3 | 2.2 ± 0.5 \| 8.1 ± 0.6 | 23.0 ± 1.4 \| 6.6 ± 3.0 | 20.7 ± 1.6 \| 33.5 ± 6.0 |
| **5** | 6 | 4 | 0.4 | GROMOS45a3 | RF | 100 | ∼110,000 | 3 | 2.5 ± 0.8 \| 8.3 ± 0.6 | 22.7 ± 1.8 \| 6.7 ± 2.7 | 19.5 ± 1.9 \| 38.1 ± 6.0 |
| **6** | 6 | 4 | 0.8 | GROMOS45a3 | RF | 100 | ∼110,000 | 3 | 2.7 ± 1.3 \| 8.1 ± 0.6 | 22.4 ± 2.0 \| 6.1 ± 3.0 | 19.5 ± 1.3 \| 32.5 ± 4.5 |
| **7** | 6 | 4 | 0 | GROMOS54a7 | RF | 100 | ∼110,000 | 1 | 3.8 ± 3.0 \| 8.6 ± 1.0 | 22.6 ± 2.1 \| 7.7 ± 2.3 | 19.9 ± 1.2 \| 41.8 ± 10.2 |
| **8** | 6 | 4 | 0.05 | GROMOS54a7 | RF | 100 | ∼110,000 | 1 | 4.3 ± 2.5 \| 8.0 ± 0.6 | 22.4 ± 2.4 \| 6.6 ± 4.6 | 19.0 ± 1.0 \| 34.3 ± 3.4 |
| **9** | 6 | 4 | 0.05 | GROMOS45a3 | PME | 200 | ∼110,000 | 3 | 2.8 ± 0.8 \| 7.9 ± 0.8 | 20.7 ± 3.2 \| 6.4 ± 3.7 | 20.9 ± 3.2 \| 29.0 ± 3.7 |
| **10** | 6 | 4 | 0.05 | GROMOS54a7 | PME | 200 | ∼110,000 | 3 | 2.4 ± 0.5 \| 7.8 ± 0.9 | 23.1 ± 1.5 \| 9.1 ± 5.0 | 21.2 ± 2.9 \| 30.7 ± 4.0 |
| **11** | 6 | 4 | 0.05 | AMBER99SB -ILDN | PME | 200 | ∼110,000 | 3 | 2.2 ± 0.4 \| NA | 21.4 ± 2.0 \| NA | 18.6 ± 0.8 \| NA |
| **12** | 6 | 4 | 0.05 | CHARMM22-CMAP | PME | 200 | ∼110,000 | 3 | 2.4 ± 0.5 \| NA | 23.1 ± 1.3 \| NA | 18.6 ± 0.6 \| NA |
| **aggregate average (GROMOS force fields only)** | | | | | | | | | 2.7 ± 1.2 \| 8.1 ± 0.8 | 22.3 ± 2.3 \| 7.0 ± 3.9 | 20.3 ± 2.4 \| 33.3 ± 5.8 |
| **Ref [31]** | ∞^-1^ | 1 | 0 | GROMOS45a3 | RF | 110 | ∼14,000 | 5 | 3.1 ± 1.4 \| 7.3 ± 0.9 | 21.8 ± 3.2 \| 12.1 ± 5.1 | 19.3 ± 1.4 \| 29.3 ± 5.3 |
